# I am my Sister’s Keeper: A Community-Engaged Research Project to Explore Perceptions of Hereditary Cancer Risk Assessment among Black Women in the Mountain West

**DOI:** 10.21203/rs.3.rs-10543530/v1

**Published:** 2026-08-25

**Authors:** Crystal Y. Lumpkins, Paula Smith, April Law, Olivia Webster, Whitney F. Maxwell, Sandra Buabeng, Kamisha L. Johnson-Davis, Cathy Wolfsfeld, Kimberly A. Kaphingst

**Affiliations:** 1 University of Utah, USA; 2 University of Utah Health, USA; 3 Huntsman Cancer Institute, USA; 4 Young Survival Coalition, USA

## Abstract

Black American (BA) women continue to experience cancer disparities in the US. These disparities warrant innovative ways to prevent cancer. Hereditary Cancer Risk Assessment (HCRA) is a cancer prevention approach that is available but not well known among BA. This pilot study aimed to explore attitudes, perceptions, and beliefs about cancer and HCRA among BA women in the Mountain West and to inform a future health promotion trial. A community-based participatory research approach was employed to engage with predominately Black organizations that have served the region for several years. Women (N=24) participated in one of five focus groups between September 2023 and April 2024. In a pre-survey, nearly half of participants responded ‘yes’ to a question about whether they were at risk for cancer; however, the majority responded that they had never talked to a medical provider about an HCRA akin to a family health history. Focus group discussions were guided by health behavior and communication theory but grounded in participants’ cancer experiences. Themes included: (1) Limited Awareness about HCRA (2) Centering Faith When Experiencing Cancer; (3) Social Support when Experiencing Cancer (4) Limited Control to address Cancer Risk Factors (particularly Breast Cancer), and (5) Racialized Healthcare Experiences. Black organizations that are community-service oriented and accessible in these communities are well-positioned to serve as trusted conduits of HCRA information dissemination among BA women in the Mountain West. Finally, more must be explored in HCRA and cancer prevention promotion to increase cancer screening and reduce inequities among BA women.

## 1.1 Introduction

Despite progress in reducing cancer-related deaths in the United States, Black Americans (BA) (African American and Black immigrants) still face a higher cancer burden across the continuum (prevention, diagnosis, treatment and survivorship) compared to other racial/ethnic groups (Siegel, et al., 2026; Centers for Disease Control and Prevention, 2025; Islami, et al., 2024). One-third of this population will face a cancer diagnosis, and one-fifth are likely to die from it (Saka, et al., 2025; American Cancer Society, 2024). BA women, a sub-population, continue to experience cancer disparities across multiple organ sites. In particular, breast and other reproductive cancers, including cervical and ovarian cancers, are of public health concern (Saka et al., 2025) (Huo & Olopade, 2007) For example, although breast cancer screening is recommended for women 40 to 74 (U.S. Preventive Services Task Force, 2024), this guideline may be insufficient for BA women, who are more likely to be diagnosed with breast cancer at a younger age than White women (Xu, et al., 2024). Further, BA women are more likely to be diagnosed with triple negative breast cancer (TNBC), an aggressive form of breast cancer that lacks targeted therapies (Amirikia et al., 2011). Nationally, BA women overall also had lower survivorship rates compared to White women (92% White women vs. 83% BA women) (Ademuyiwa et al., 2017). Thus, greater efforts are needed to reduce breast and other cancer incidence and mortality among BA women (American Cancer Society, 2024). In the Mountain West these cancers reflect other regional and national statistics and disparities (Centers for Disease Control and Prevention, 2026; National Cancer Institute & Centers for Disease Control and Prevention, 2026). Biological factors explain cancer outcomes among this population in part, however, psycho-social and other factors related to geography and/or environment may also contribute to disparities (Barreto-Coelho, 2019). In particular, social determinants of health impact cancer outcomes (Alcaraz et al., 2020) through access to care, limited awareness, lack of cancer provider discussion (Reid et al., 2020; Cragun et al., 2017) and psycho-social factors including emotional and social support (Jernigan et al., 2001). These factors specifically, among Black immigrant women, may be shaped by structural or social barriers including lack of trust in the medical system, immigration status, food and housing insecurity, and living in geographic areas with limited access to health services (Coughlin, 2019).

Combating cancer disparities among BA women therefore requires a multi-pronged approach that is inclusive of addressing cancer disparities through pinpointing both psycho-social and systematic barriers to cancer care (Cho et al., 2021). Utilization of HCRA may be a helpful approach in determining the probability for cancer risk, particularly if there is a personal or family health history of cancer risk. Gomy and Diz (2013) describe HCRA as part of a multi-step process and how it may assist in decision making about cancer risk. “*It includes genetic counseling, testing and management of at-risk individuals so that they can make well-informed choices about cancer surveillance, surgical treatment and chemo-preventive measures, including biomolecular cancer therapies, providing the best path for genetic counseling and testing*. (p.1)”. While improving access about HCRA may positively impact cancer outcomes among BA, this assessment and subsequent genetic counseling and testing services, awareness is limited among both racial/ethnic minority groups (Muessig et al., 2022; Hann et al., 2017; Sheppard et al., 2014) and the capacity for information dissemination and education are limited overall. Community and membership organizations within Black communities have shown as effective information disseminators and educators for cancer prevention (McNeil, 2018). Further, organizations within these communities are positioned to be important health promoters (Woodard et al., 2022) and thus advocates of HCRA and other pertinent cancer risk and prevention information. Many of the cancer health risk assessment information disparities among BA are indicative of larger information inequality issues about cancer prevention tools that consider family health history in determining cancer risk. Because of limited and emerging research on health promotion about and barriers to HCRA uptake among BA women in the Mountain West, the aims of the study were to: (1) explore existing perceptions, beliefs, awareness and cancer experiences among BA women; and (2) explore what role, if any, community and member organizations had in HCRA information dissemination and education.

## 2.1 Methods

### 2.1 Theoretical framework

A mixed-methods approach was used, where a theoretical framework guided the discussion questions and grounded theory informed how phenomena were interpreted by participants. Cancer experiences and responses to questions among participants expressed through conversations were used as building blocks of theory (Creswell, 2007) and provided a guide to understand how BA women perceived cancer. This approach included the adoption of constructs (Mitchell, 2014 and Charmaz, 2014) from (1) the Theory of Planned Behavior (attitudes toward cancer and cancer screening; subjective norms and perceived control of cancer screening) (Boslaugh, 2022) (Ajzen, 1991); (2) Social Capital Theory concepts of trust, reciprocity, and civic engagement within organizational environments (Putnam, 1995; Stephens, et al., 2004); and (3) McGuire’s Input-Output communication framework (McGuire, 1989).

These constructs aimed to capture attitudes toward cancer and HCRA, subjective norms or what they thought their significant peers thought about these factors, perceived control of cancer outcome and HCRA (Boslaugh, 2022) and perceptions about the affiliation and capacity an organization has in promoting this type of health information (See table 1). McGuire’s Input-Output framework was used as a guide to ask participants what they thought about cancer prevention messages that were delivered by the FBO or CBO. The focus of these questions was to ask participants what they thought about the source, message, channel and receiver of cancer and HCRA related messages.

### 2.2 Study design and approach

A community-based participatory research (CBPR) approach was employed and included aims to build capacity with a Black Greek Letter (membership) Organization (BGLO) and to explore perceptions about HCRA and cancer experiences among BA women in the state of Utah (Mountain West region). The study was approved by the University of Utah Institutional Review Board (Protocol #00170994). A Community Action Team (CAT) was created in November 2022 and comprised of members of the Physical and Mental Health Committee of the Greater Salt Lake City Alumnae Chapter of Delta Sigma Theta, Sorority, Inc., a BGLO, and representatives from other predominately Faith and Community-Based organizations (Calvary Baptist Church, Second Baptist Church, Black Physicians of Utah, Mothers Shut In). CAT members helped guide several parts of the research design, including study questions, recruitment, and data collection. Focus group discussions were conducted between September 2023 and April 2024 in Salt Lake City and Ogden, Utah. The study was a qualitative exploratory design where 8 CAT members and 3 research staff members co-developed the focus group moderator’s guide, recruitment strategy and data collection; research staff subsequently executed qualitative data analysis and reporting. The sample was purposive, where BA women were recruited from the BGLO and other BA networks within the Greater Salt Lake City area. Recruitment activities included a Juneteenth celebration in Ogden, Utah, church services (morning, evening and weekend services – bible study, worship service, women’s mission) and a REDCap (Research Electronic Data Capture) (Harris et al., 2009) survey disseminated to residents at a graduate housing site on the University of Utah campus.

### 2.3 Study team

Team members consisted of the principal investigator, co-investigators, research assistants, and CAT members. CAT members were from the BGLO and who received IRB training . The principal investigator self-identifies as an African American woman; co-investigators self-identify as White women and are a social scientist (KK) and a genetic counselor (WM). The research assistants who worked on the project were white (AL), African American (KD) and bi-racial (Black and Latina) (OW). CAT members all self-identified as from the African Diaspora (SB, KJD, PW, CW, PS). The Principal Investigator facilitated all focus groups.

### 2.4 Study setting

All focus group discussions occurred either in a church conference/meeting room or in a private space within a community building to reinforce privacy. Participants were instructed at the beginning of the session that they needed to maintain the confidentiality of the other participants and their responses. Transcripts were de-identified, and participant contributions were not linked to their identities. All focus groups were approximately 90–120 minutes in length, including time for registration, consent and meal service.

### 2.5 Data collection

Inclusion criteria for the study included self-identification as: (1) Black, (2) a woman, (3) at least 18 years old (4) English speaking, and (5) a resident of the Greater Salt Lake City area. Prior to the focus group, participants were asked demographic questions that included these eligibility criteria and additional cancer-related questions (risk for cancer, previous cancer screening ). There were two focus groups where one individual self-identified as *other* than BA; the pre-survey data was excluded however they were present in the focus groups after being consented. Data collection commenced with the first focus group on September 12, 2023, and concluded with the final focus group on April 13, 2024.

### 2.6 Participant characteristics (See table 2)

The majority of focus group participants (N=24) had some college or higher education (85%) and insurance (88%). Nearly half indicated that they were at high risk for cancer (46%) when asked “*Do you have a reason to believe you are at high risk for cancer?*”; however, the majority (88%) responded that they had *not* talked to a medical provider about a Hereditary Cancer Risk Assessment (HCRA akin to family health history, genetic counseling or testing).

### 2.7 Data analysis

Data were analyzed using the constant comparison approach (Glaser, 2008) to pinpoint themes across focus group data. Three primary coders independently read and subsequently coded transcripts. The coders then came together to compare and contrast codes and themes that emerged. One additional coder’s analysis was added because of content expertise in genetic counseling. This coder, after completing independent reading and analysis, joined two of the original primary coders in a meeting to determine codes and themes across the data. A final review across all coders (4) and data was combined into a single Excel spreadsheet to organize the data.

## 3. Results

### 3.1 Themes (See table 3)

Themes and a sub-theme relating to cancer experience and HCRA perceptions included: (1) Limited Awareness about HCRA; (sub-theme) Importance of Family Cancer Communication (2) Centering Faith when Experiencing Cancer (3) Social Support when Experiencing Cancer (4) Limited Control to address Cancer Risk Factors (particularly Breast Cancer) and (5) Racialized Healthcare Experiences.

#### 3.2.1 *Theme 1 -* Limited Awareness and Education about HCRA (Hereditary Cancer Risk Assessment)

Across all focus groups, participants were unaware of HCRA and its utility in estimating cancer risk. In some ways, limited awareness served as a barrier to taking a step to get a cancer risk assessment and/or subsequent genetic testing.

“I donť think I have enough information to know if I should participate or not, I donť think I do.” (Participant, Site A)

“If everything is fine, I’m probably not thinking twice” (about genetic testing) (Participant, Site C)

“When I was in college, we did sickle cell tests. So, is that considered genetic testing? Now that I was OK with, but you know to test, to see if I have a cancer gene, I'm just not sure if I want to do that because if I have it…” (Participant, Site A)

“I'm so happy to open to doing that because I have never heard about that. So, if thaťs actually a thing, I'm jumping on that.” (Participant, Site C)

“This is my first time hearing about.” (Participant, Site C)

This gap in communication about HCRA from medical or clinical personnel also emphasized that while these tools are available the option to receive information about cancer risk assessment was not provided.

When participants were asked what they thought about their community organization sponsoring or promoting cancer prevention or HCRA information, the importance of family cancer communication emerged as a sub-theme.

“But in cases where the families are able to like sponsor the whole thing and having hearing like you don’t even hear about it except when the person died in the process. I mean, no, you donť hear about it.” (Participant, Site C).

“You know more awareness and like I have custody of my grandchildren. So, I think about their... You know where I had urinary cancer. You know, I want them to get help and get the testings and other research for them.” (Participant, Site B)

#### 3.2.2 *Theme 2 –* Centering Faith When Experiencing Cancer

Participants were primarily members or affiliates of Faith Based Organizations (FBOs), and Community-based organizations (CBOs). In all focus group discussions, the element of faith and acceptance emerged as an avenue to cope with experiencing cancer personally or seeing a loved one experience cancer (e.g., cancer diagnosis, treatment, survivorship). This theme was tied to how participants formed attitudes and opinions toward cancer and rationale for cancer risk assessment tools in general.

“I talked to God about it, and I told Him I got cancer. Cancer donť have me. And so, I didnť stop.” (Participant, Site A)

“Do what the doctor say while you’re praying. And as a Christian, I believe in prayer.” (Participant, Site B)

“God can work through your doctor.” (Participant, Site C)

“If you're getting your pastors to pray for you, you can also still get your physician to do what you do on the medical side of things while you deal with the spiritual side of things that end up.” (Participant, Site C)

For most participants, faith was expressed as a protective factor; faith in God was a motivator to be proactive in their health. Some sentiments expressed in the focus groups, however, were passive and resembled fatalism in that God would take care of them and that there wasn’t a need to do this type of preventive screening.

“But you know, the thing too is that God will take care of you. I donť need to do this. Yeah, I donť need to do that because God, got, me. So, what do you, you know what I mean?” (Participant, Site A)

“As far as I see it, the way it is in Nigeria is the, the religion is here and medical is here (motions with her hands). Like religion comes before medical.” (Participant, Site C)

#### 3.2.3 *Theme 3 -* Social Support when Experiencing Cancer

Providing social support or needing support when experiencing cancer as an individual or as a family/church or organization member was a common theme throughout and re-emphasized the importance of affiliation with an FBO or CBO. These entities were seen as critical when members encountered cancer experiences and/or received cancer information.

“I donť have a lot of people to come. I have everybody in the world that I help, but I donť have very many personal people to communicate to, you know what I'm saying?” (Participant, Site A)

“I was always trying to help, always trying to be like the cheerleader offering my services, whatever they needed. So, for me, I just hope that like with cancer, like, people get the support like the cheerleaders, they need like, like that kind of helps.” (Participant, Site B)

“I try to encourage them. To go ahead and get whatever test is necessary.” (Participant, Site B)

#### 3.2.4 *Theme 4 -* Limited Control to address Cancer Risk Factors (particularly Breast Cancer)

The Theory of Planned Behavior (TPB) served as part of the framework to guide exploration of HCRA perceptions among BA women. Within this theory, three constructs, attitudes toward the behavior, subjective norms and perceived control of completing a behavior are posed to be predictors of a behavior change (Boslaugh, 2022; Ajzen, 1991). Of the three TPB constructs explored, perceived control (self-efficacy) to complete a behavior (Ajzen, 2002) was thought to be limited. Participants in the group discussions thought about control in cancer experiences as a loss because they had limited information and knowledge to act or complete a cancer risk assessment. More specifically in terms of HCRA, well-known ‘others’ (not peers) who could bring this information to the forefront in the public’s eye would make a difference. There was a sense that through emphasis from a well-known person or celebrity, cancer risk assessment could be something that could be completed.

“We donť know enough about all cancers, you know, to where different ones that are highlighted. Breast cancer gets huge walks and everything else about it. You can get this. You can get that. You donť really hear or talk about or have the resources or know where to go for all the other ones.” (Participant, Site B)

“Should I get genetic testing to see if I'm the candidate that there is no genetic testing right now for pancreatic cancer? One of the ones thaťs kind of hard.” (Participant, Site B)

#### 3.2.5 *Theme 5*: Racialized Healthcare Experiences

Participants across all focus groups expressed experiences that may hinder them from HCRA and other cancer prevention screenings because of negative and/or racialized healthcare encounters and limited communication to discuss cancer risk and prevention. While some participant comments were overtly expressed as racist or discriminatory, participants from other focus groups described their experiences as covert; other words or phrases akin to ‘exclusion,’ ‘other people,’ ‘not me’ within the context of healthcare and HCRA access were interpreted as negative/racialized healthcare experiences.

“And so, we know that there's researches that are being done out there that are not being brought to us, to our communities as a black American.” (Participant, Site A)

“Me, I would like to see more doctors that are for the black women, for us because I'm like, always looking for just another opinion or someone that knows our body and knows what we go through.” (Participant, Site B)

“I just know that you go to a hospital, doctors treat you for the chemo and all this stuff, but it seems like they're treating cancer like iťs like the same for like everybody, and they just clump it together. Like, you know what, like they do, like race it, like, not all black people are just fully black or African. They can be from like Jamaica or they can be from, you know, South America. They can be all different.” (Participant, Site B)

“As a black woman in America, I need people to step it up and give me the assistance that I need, that I see everybody else is receiving. Donť make it hard for me to get to a doctor when I know that one other person is like the next person next to me can go right down the street and get the assistance that they need. I need you to care just as much as you care for that other person that you care for me too.” (Participant, Site B)

## 4. Conclusion and future directions

This study was part of an effort to build a cancer prevention education partnership with predominately African American organizations in the Mountain West. The study aims for this project were to explore perceptions, attitudes and beliefs about HCRA among BA women and how this may serve as important building blocks for cancer prevention education programming within FBOs and CBOs in the region. Through qualitative inquiry, the study research team and CAT (Community Advisory Team) found there to be limited awareness among this pilot sample; however, faith and social support emerged as critical factors when discussing cancer-related topics. Family members, at least among some participants, were seen as important communicators of this type of information. Some participants also saw the value of their organization as sponsors and or communicators of HCRA. Barriers included limited control with HCRA and other negative healthcare experiences.

The lack of awareness and other systematic factors have created barriers to genetic services and underutilization among BA and other racial/ethnic groups (Cragun et al., 2017; Huo et al., 2007). Increased physician communication to recommend and/or communicate genetic services to BA and other racial/ethnic women (Reid, 2020), and identification of how to reach marginalized groups about genetic services and tools may improve cancer outcomes (DeSantis et al., 2019; Huo et al., 2007). While some in the sample saw their faith as a protective factor, others in the sample were more fatalistic and expressed a faith in God and that he would take care of them. Racialized/negative healthcare systems and negative cancer experiences posed as barriers to HCRA information and uptake. Limited awareness of HCRA and specific psycho-social barriers to HCRA call for more concentrated efforts to address gaps in cancer communication among BA women that may mitigate cancer disparities. These efforts may also be beneficial to pinpointing regional disparities or help reduce inequities among BA women in less targeted areas similar to the Mountain West.

The present study is one that builds on existing literature of exploration of perceptions, attitudes, and beliefs about cancer, cancer risk, and HCRA among BA, particularly Black immigrant women(Cudjoe et al., 2019). To our knowledge, this is one of the few studies dedicated to exploring perceptions among BA individuals affiliated with affinity organizations which could be instrumental in information dissemination and promotion of HCRA risk communication. The sample was purposive where recruitment was conducted with both the study team and CAT (Community Advisory Team members) within specific and selected communities. While the partner organization, the Greater Salt Lake Alumnae Chapter of Delta Sigma Theta, Sorority Inc. served as the primary partner organization, most of the focus groups were held at an FBO site except for one. In addition, there was one focus group that would be best described as a group discussion where there were only 3 individuals who participated. Finally, there were two focus group where an individual self-identified as *other* than BA; the pre-survey data was excluded however they were present in the focus groups after being consented by the study team. In efforts to maintain a rapport with community organizations, the Principal Investigator allowed the individual to remain out of respect for the partnership and to reduce any harm. Analysis of data included pinpointing this individual contribution and determining how these narratives either inflated or confounded any results.

The Black Greek Letter Organization (BGLO), the Greater Salt Lake Alumnae Chapter of Delta Sigma Theta Sorority, Inc., who partnered with the research team in this study, has had dedicated programming to advance the overall health and wellness of BA populations and women overall. Community organizations like these in the region are also well positioned to serve as trusted conduits of health communication and health education. In addition, these organizations, with training and focus on infrastructure, may also be positioned along with other organizations (Black Physicians of Utah or BPOU) to provide medical referrals and also access to follow up and medical care. The Co-Principal Investigator from the partner organization is also a nurse who served within the community and the region for 40+ years.

Given existing cancer and other disease-oriented/health disproportions among young and older BA women within the Mountain West, priority should be placed on building capacity that becomes building blocks to fruitful partnerships with community organizations concerning HCRA and other cancer prevention and promotion. Through these partnerships and discovery of similar networks, HCRA and cancer prevention information dissemination may be instrumental and a step to increasing prevention to reduce cancer inequities among BA.

## Figures and Tables

**Table 1:** Focus Group Theoretical Framework

Grounded Theory/Theoretical Framework	
Semi-Structured Interview Guide (Sample) Questions	**Constructs**

**Theory of Planned Behavior (Azjen, 1991)**	

When you hear the word cancer, what comes to mind?	**Attitude Toward Cancer**
What comes to mind when you think about genetic counseling and testing?	**Attitude Toward Genetic counseling and Testing/HCRA**
Do you think people are aware of genetic counseling and testing as it relates to cancer?	

What would you think about genetic counseling and testing if it could predict the risk of cancer in your family?	**Subjective Norms**

How does cost figure into the decision of whether or not to get genetic counseling and genetic testing?	**Perceived Control for Genetic Counseling and Testing/HCRA**

**Social Capital Theory (Putnam, 1995)**	

If you have received information from a Community Health Worker or Lay Health Advisor (within your FBO/CBO), how was this experience?	**Reciprocity/Trust**

**McGuire’s Classic Input-Output Framework (McGuire, 1989)**	

Who would you consider a trusted person within your community to share genetic counseling and genetic testing messages?	**Source**
What strategy do you think would be most effective in getting genetic counseling and genetic testing messages across to patients?	**Message**
What style of message delivery seems best?	**Channel**

**Table 2:** Sample Demographic Characteristics

**Sample (n = 25)**	
**Characteristic**	Total n (%)
**Age**	
**18–24**	0 (0%)
**25–34**	4 (16%)
**35–44**	4 (16%)
**45–54**	2 (8%)
**55+**	16 (64%)
**Gender**	
**Male**	0 (0%)
**Female**	25 (100%)
**Other**	0 (0%)
**Current Living Situation**	
**Married**	10 (40%)
**Living w/ a Partner**	1 (4%)
**Divorced**	5 (20%)
**Single**	4 (16%)
**Separated/Widowed**	4 (16%)
**Never Married**	1 (4%)
**Other**	1 (4%)
**Education**	
**Less than High School (12^th^ Grade)**	2 (8%)
**High School/General Education Development (GED)**	2 (8%)
**Some College/Technical**	7 (26.9%)
**Associate’s degree/Training**	2 (8%)
**4-year College/4-year Training**	5 (20%)
**Post Baccalaureate/Graduate**	8 (32%)
**Other Training**	0 (0%)
**Employment**	
**Full Time**	12 (48%)
**Part-Time**	4 (16%)
**Out of work for more than 1 year**	0 (0%)
**Out of work for less than 1 year**	2 (8%)
**Stay at home**	2 (8%)
**Student**	0 (0%)
**Retired**	6 (24%)
**Unable to work**	0 (0%)
**Total Annual Household Income**	
**Less than $10,000**	2 (8%)
**$10,000- $29,999**	9 (36%)
**$30,000- $49,999**	2 (8%)
**$50,000- $69,999**	3 (12%)
**$70,000- $99,999**	4 (16%)
**$100,000- and above**	5 (20%)
**Ethnic/Racial Background**	
**White**	1 (4%)
**Black/African American**	23 (92%)
**Hispanic/Latino**	0 (0%)
**Asian**	0 (0%)
**American Indian/Alaskan Native**	2 (8%)
**Native Hawaiian/Pacific Islander**	0 (0%)
**Other**	1 (4%)

**Table 3:** Sample Quotes from Focus Group Discussions

Themes	Site	Participant Quotes

1. Limited Awareness about HCRA	A	“I don’t think I have enough information to know if I should participate or not; I don’t think I do.”
	C	“If everything is fine, I’m probably not thinking twice” (about genetic testing)
	A	“When I was in college, we did sickle cell tests. So, is that considered genetic testing? Now that I was OK with, but you know to test, to see if I have a cancer gene, I'm just not sure if I want to do that because if I have it...”
	C	“I'm so happy to open to doing that because I have never heard about that. So, if that’s actually a thing, I'm jumping on that.”
	C	“This is my first time hearing about.”
2. Faith/Acceptance in Experiencing Cancer	C	“God can work through your doctor.”
	A	“I talked to God about it, and I told Him I got cancer. Cancer don’t have me. And so, I didn’t stop.”
	B	“Do what the doctor say while you’re praying. And as a Christian, I believe in prayer.”
	C	“If you're getting your pastors to pray for you, you can also still get your physician to do what you do on the medical side of things while you deal with the spiritual side of things that end up.”
	A	“But you know, the thing too is that God will take care of you. I don’t need to do this. Yeah, I don’t need to do that because God —got —me. So, what do you, you know what I mean?”
3. Social Support when Experiencing Cancer	A	“I don’t have a lot of people to come. I have everybody in the world that I help, but I don’t have very many personal people to communicate to, you know what I'm saying?”
	B	“I was always trying to help, always trying to be like the cheerleader offering my services, whatever they needed. So, for me, I just hope that like with cancer, like, people get the support like the cheerleaders, they need like, like that kind of helps.”
	B	“I try to encourage them to go ahead and get whatever test is necessary.”
4. Limited Control to address Cancer Risk Factors (particularly Breast Cancer)	B	“We don’t know enough about all cancers, you know, to where different ones that highlighted. Breast cancer gets huge walks and everything else about it. You can get this. You can get that. You don’t really hear or talk about or have the resources or know where to go for all the other ones.”
	B	“Should I get genetic testing to see if I'm the candidate that there is no genetic testing on thing right now for pancreatic cancer? One of the ones that’s kind of hard.”
5. Racialized Healthcare Experiences	A	“And so, we know that there's researches that are being done out there that are not being brought to us, to our communities as a black American.”
	B	“Me, I would like to see more doctors that are for the black women, for us because I'm like, always looking for just another opinion or someone that knows our body and knows what we go through.”
	B	“I just know that you go to a hospital, doctors treat you for the chemo and all this stuff, but it seems like they're treating cancer like it’s like the same for like everybody, and they just clump it together. Like, you know what, like they do, like race it, like, not all black people are just fully black or African. They can be from like Jamaica or they can be from, you know, South America. They can be all different.”
	B	“As a black woman in America, I need people to step it up and give me the assistance that I need, that I see everybody else is receiving. Don’t make it hard for me to get to a doctor when I know that one other person is like the next person next to me can go right down the street and get the assistance that they need. I need you to care just as much as you care for that other person that you care for me too.”
a. Importance of Family Cancer Communication	B	“You know more awareness and like I have custody of my grandchildren. So, I think about their... You know, where I had urinary cancer. You know, I want them to get help and get the testings and other research for them.”
	C	“But in cases where the families are able to like sponsor the whole thing and having hearing like you don’t even hear about it except when the person died in the process. I mean, no, you don’t hear about it.”

## Data Availability

De-identified transcripts and surveys may be requested by contacting the corresponding author.

## References

[R1] Ademuyiwa F. O., Tao Y., Luo J., Weilbaecher K., & Ma C. X. (2017). Differences in the mutational landscape of triple-negative breast cancer in African Americans and Caucasians. Breast Cancer Res Treat, 161(3), 491–499. 10.1007/s10549-016-4062-y 27915434 PMC5243212

[R2] Ajzen I. (1991). The theory of planned behavior. Organizational behavior and human decision processes, 50(2), 179–211. 10.1016/0749-5978(91)90020-T

[R3] Ajzen I. (2002). Perceived Behavioral Control, Self-Efficacy, Locus of Control, and the Theory of Planned Behavior. Journal of Applied Social Psychology, 32(4), 665–683. 10.1111/j.1559-1816.2002.tb00236.x

[R4] Alcaraz K. I., Wiedt T. L., Daniels E. C., Yabroff K. R., Guerra C. E., & Wender R. C. (2020). Understanding and addressing social determinants to advance cancer health equity in the United States: A blueprint for practice, research, and policy. CA Cancer J Clin, 70(1), 31–46. 10.3322/caac.21586 31661164

[R5] Amirikia K. C., Mills P., Bush J., & Newman L. A. (2011). Higher Population-Based Incidence Rates of Triple-Negative Breast Cancer Among Young African-American Women Implications for Breast Cancer Screening Recommendations. Cancer, 117(12), 2747–2753. 10.1002/cncr.25862 21656753 PMC3461243

[R6] Barreto-Coelho P., Cerbon D., Schlumbrecht M., Parra C. M., Hurley J., & George S. H. L. (2019). Differences in breast cancer outcomes amongst Black US-born and Caribbean-born immigrants. Breast Cancer Res Treat, 178(2), 433–440. 10.1007/s10549-019-05403-9 31414243 PMC7039732

[R7] Boslaugh S. E. (2022). Theory of planned behavior. In E. R. S. S. S. a. Humanities. (Ed.).

[R8] Charmaz K. (2014). Constructing grounded theory (2nd . ed.). London, Thousand Oaks, Calif. : Sage.

[R9] Cho B., Han Y., Lian M., Colditz G. A., Weber J. D., Ma C., & Liu Y. (2021). Evaluation of racial/ethnic differences in treatment and mortality among women with triple-negative breast cancer. JAMA oncology, 7(7), 1016–1023. 33983438 10.1001/jamaoncol.2021.1254 PMC8120441

[R10] Coughlin S. S. (2019). Social determinants of breast cancer risk, stage, and survival. Breast Cancer Res Treat, 177(3), 537–548. 10.1007/s10549-019-05340-7 31270761

[R11] Cragun D., Weidner A., Lewis C., Bonner D., Kim J., Vadaparampil S. T., & Pal T. (2017). Racial disparities in BRCA testing and cancer risk management across a population-based sample of young breast cancer survivors. Cancer, 123(13), 2497–2505. 10.1002/cncr.30621 28182268 PMC5474124

[R12] Creswell J. W. (2007). Qualitative inquiry & research design : choosing among five approaches (2nd . ed.). Thousand Oaks : Sage Publications.

[R13] Cudjoe J., Turkson-Ocran R.-A., Ezeigwe A. K., Commodore-Mensah Y., Nkimbeng M., & Han H.-R. (2019). Recruiting African Immigrant Women for Community-Based Cancer Prevention Studies: Lessons Learned from the AfroPap Study. Journal of community health, 44(5), 1019–1026. 10.1007/s10900-019-00677-y 31102117 PMC6708449

[R14] DeSantis C. E., Miller K. D., Goding Sauer A., Jemal A., & Siegel R. L. (2019). Cancer statistics for African Americans, 2019. CA Cancer J Clin, 69(3), 211–233. 10.3322/caac.21555 30762872

[R15] Force, U. S. P. S. T. (2024). Final recommendation statement: Breast cancer: Screening. https://www.uspreventiveservicestaskforce.org/uspstf/recommendation/breast-cancer-screening

[R16] Glaser B. G. (2008). The Constant Comparative Method of Qualitative Analysis. The grounded theory review, 7(3).

[R17] Greater Salt Lake Alumnae Chapter of Delta Sigma Theta Sorority, I. Utah DST GSLAC. Retrieved 6/24/25 from https://www.utahdst.org/

[R18] Hann K. E. J., Freeman M., Fraser L., Waller J., Sanderson S. C., Rahman B., Side L., Gessler S., & Lanceley A. (2017). Awareness, knowledge, perceptions, and attitudes towards genetic testing for cancer risk among ethnic minority groups: a systematic review. BMC public health, 17(1), 503–503. 10.1186/s12889-017-4375-8 28545429 PMC5445407

[R19] Harris P. A., Taylor R., Thielke R., Payne J., Gonzalez N., & Conde J. G. (2009). Research electronic data capture (REDCap)—A metadata-driven methodology and workflow process for providing translational research informatics support. Journal of Biomedical Informatics, 42(2), 377–381. 10.1016/j.jbi.2008.08.010 18929686 PMC2700030

[R20] Huo D., & Olopade O. I. (2007). Genetic Testing in Diverse Populations: Are Researchers Doing Enough to Get Out the Correct Message? JAMA : the journal of the American Medical Association, 298(24), 2910–2911. 10.1001/jama.298.24.2910 18159061

[R21] Islami F., Baeker Bispo J., Lee H., Wiese D., Yabroff K. R., Bandi P., Sloan K., Patel A. V., Daniels E. C., Kamal A. H., Guerra C. E., Dahut W. L., & Jemal A. (2024). American Cancer Society’s report on the status of cancer disparities in the United States, 2023. CA Cancer J Clin, 74(2), 136–166. 10.3322/caac.21812 37962495

[R22] Jernigan J. C., Trauth J. M., Neal-Ferguson D., & Cartier-Ulrich C. (2001). Factors That Influence Cancer Screening in Older African American Men and Women: Focus Group Findings. Fam Community Health, 24(3), 27–33. 10.1097/00003727-200110000-00005 11563942

[R23] McGuire W. J. (1989). Theoretical foundations of campaigns. In Public Communication Campaigns (2nd ed.). Sage.

[R24] Mitchell D. (2014). Advancing Grounded Theory: Using Theoretical Frameworks within Grounded Theory Studies. Qualitative report. 10.46743/2160-3715/2014.1014

[R25] Muessig K. R., Zepp J. M., Keast E., Shuster E. E., Reyes A. A., Arnold B., Ingphakorn C., Gilmore M. J., Kauffman T. L., Hunter J. E., Knerr S., Feigelson H. S., & Goddard K. A. B. (2022). Retrospective assessment of barriers and access to genetic services for hereditary cancer syndromes in an integrated health care delivery system. Hered Cancer Clin Pract, 20(1), 7–10. 10.1186/s13053-022-00213-5 35144679 PMC8832647

[R26] Prevention, C. f. D. C. a. (2025). Cancer and African American people. https://www.cdc.gov/cancer/health-equity/african-american.html

[R27] Prevention, C. f. D. C. a. (2026). CDC WONDER: Data request page. https://wonder.cdc.gov/cancermort-v2022_SR.html

[R28] Prevention, N. C. I. C. f. D. C. a. (2026). State Cancer Profiles: Incidence rates. https://statecancerprofiles.cancer.gov/incidencerates/index.php?stateFIPS=00&areatype=state&cancer=001&race=00&sex=0&age=001&year=0&type=incd&sortVariableName=rate&sortOrder=default&output=0#results

[R29] Putnam R. D. (1995). Bowling Alone: America’s Declining Social Capital. Journal of Democracy, 6(1), 65–78.

[R30] Reid S., Cragun D., Tezak A., Weidner A., Moore J., Mayer I. A., Shu X. o., Ye F., Fan R., Vadaparampil S., & Pal T. (2020). Disparities in BRCA counseling across providers in a diverse population of young breast cancer survivors. Genet Med, 22(6), 1088–1093. 10.1038/s41436-020-0762-0 32066870 PMC7275890

[R31] Saka A. H., Giaquinto A. N., McCullough L. E., Tossas K. Y., Star J., Jemal A., & Siegel R. L. (2025). Cancer statistics for African American and Black people, 2025. CA Cancer J Clin, 75(2), 111–140. 10.3322/caac.21874 39976243 PMC11929131

[R32] Sheppard V. B., Graves K. D., Christopher J., Hurtado-de-Mendoza A., Talley C., & Williams K. P. (2014). African American Women’s Limited Knowledge and Experiences with Genetic Counseling for Hereditary Breast Cancer. J Genet Couns, 23(3), 311–322. 10.1007/s10897-013-9663-6 24186304 PMC4255465

[R33] Siegel R. L., Kratzer T. B., Wagle N. S., Sung H., & Jemal A. (2026). Cancer statistics, 2026. CA: a cancer journal for clinicians, 76(e70043). 41528114 10.3322/caac.70043 PMC12798275

[R34] Society, A. C. (2024). Breast cancer Facts & Figures 2024–2026. https://www.cancer.org/content/dam/cancer-org/research/cancer-facts-and-statistics/breast-cancer-facts-and-figures/2024/breast-cancer-facts-and-figures-2024.pdf

[R35] Stephens K. K., Rimal R. N., & Flora J. A. (2004). Expanding the reach of health campaigns: Community organizations as meta-channels for the dissemination of health information: Forty year of diffusion of innovations: utility and value in public health. J Health Commun, 9, 97–111. 14960406 10.1080/10810730490271557

[R36] Xu S., Murtagh S., Han Y., Wan F., & Toriola A. T. (2024). Breast Cancer Incidence Among US Women Aged 20 to 49 Years by Race, Stage, and Hormone Receptor Status. JAMA Netw Open, 7(1), e2353331. 10.1001/jamanetworkopen.2023.53331 38277147 PMC10818222

